# Supplementary material for: Evidence against implicit belief processing in a blindfold task
Source: PLoS One. 2023 Nov 13;18(11):e0294136. doi: 10.1371/journal.pone.0294136 (PMC10642834; doi:10.1371/journal.pone.0294136)
Supplement: S1 File — (DOCX) [file pone.0294136.s001.docx]

Supporting information

S1 Power Simulations

We simulated data with the function ‘ANOVA_design’ of the R package ‘Superpower’ version 0.1.0 (Lakens & Caldwell, 2019) using information about effect sizes (partial eta squared = 0.06, Cohen’s f = 0.25) from a previous study (El Kaddouri, Bardi, De Bremaeker, Brass, & Wiersema, 2019). Then, we calculated the Bayes Factor of our main variable of interest for this simulated data set while increasing the sample size successively in steps of 10 starting with our predefined minimal sample size of 68. We repeated this process 10,000 times, counted how often we correctly reached our preselected stopping criterion of 4, how often we mistakenly reached the stopping criterion of 0.25 (indicating no effect) and how often results remained inconclusive even for our maximal sample size of 500. This gave us information about the power of our sequential approach (simulations ran in favor of an effect in 83.96% of the cases). To control for false positives, we ran the same calculations while simulating no effect (simulations provided evidence for an effect in 5% of the cases). Also, we noted at which sample size the stopping criterion was reached in each simulation which provided information about the expected sample size (Median sample size of 78 if an effect was simulated and 72 if no effect was simulated).

S2 Experiment 1: Violation of Normality

Quantile-quantile (Q-Q) plots indicated right skewed distributions for the individual response times in the object and the action detection task that deviated from normality. After log-transformation only minor deviations from normality were observed (see S1 Fig).

**S1 Fig. Q-Q-Plots of the log-transformed IRTs in experiment 1.**


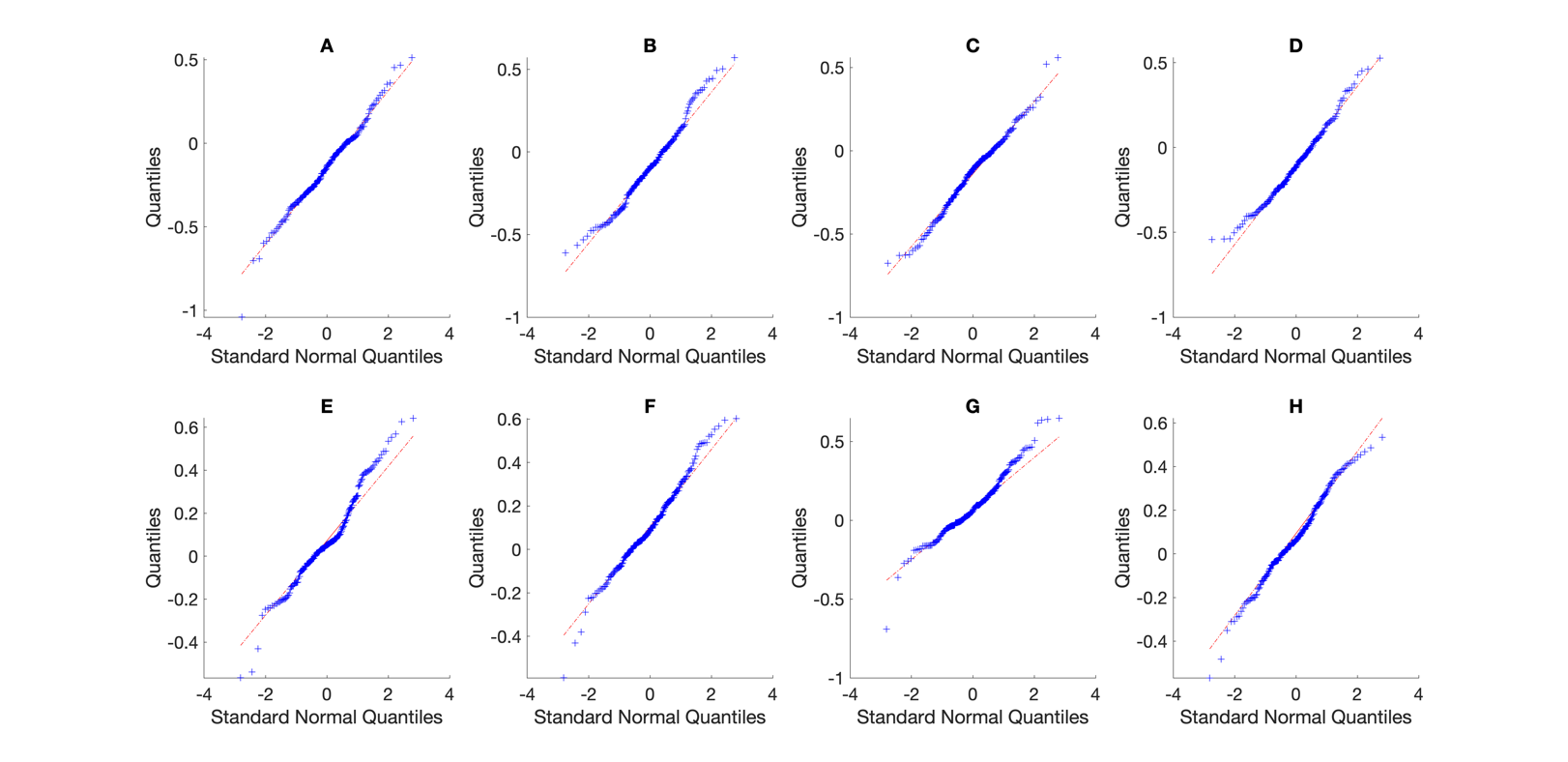


Quantiles of the log-transformed individual response times plotted against the theoretical quantile values from a normal distribution. A: log-IRTs in true belief reality congruent trials in the object detection task, B: log-IRTs in true belief reality incongruent trials in the object detection task, C: log-IRTs in false belief reality congruent trials in the object detection task, D: log-IRTs in false belief reality incongruent trials in the object detection task, E: log-IRTs in true belief reality congruent trials in the action detection task, F: log-IRTs in true belief reality incongruent trials in the action detection task, G: log-IRTs in false belief reality congruent trials in the action detection task, H: log-IRTs in false belief reality incongruent trials in the action detection task.

S3 Experiment 1: Sequential Testing

In experiment 1, the sequential testing strategy led to N=119 participants of which 90 could be included for the object detection task and 102 for the action detection task. For the object detection task, the stopping criterion was already reached for the minimal number of included participants (i.e. 68). Still, only including the data of these 68 participants did not change the results (see S1 Table). For the action detection task, the stopping criterion was first reached for 86 included participants. Since recruitment and participation in the online study was automatized, it was not stopped until data from 16 additional participants were collected. However, removing this data did not significantly alter the results (see S2 Table). S2 Fig illustrates how the Bayes Factors developed with increasing number of included participants.

**S1 Table. Bayes Factors for the model comparisons in the object detection task of exp. 1 for n=68.**

|  | Model 0 (1^st^ row) and Model 1 (2^nd^ row) | BF | Error | BF10 | Error |
| --- | --- | --- | --- | --- | --- |
| Belief | IRT ~ ID + C | 0.1110 | 0.82% | 0.1070 | 0.74% |
|  | IRT ~ ID + C + B | 26.2288 | 0.57% |  |  |
| Congruency | IRT ~ ID + B | 245.1178 | 0.47% | 236.20 | 1.00% |
|  | IRT ~ ID + B + C | 26.2288 | 0.57% |  |  |
| Belief * Congruency | IRT ~ ID + B + C | 26.2288 | 0.57% | 0.1711 | 0.86% |
|  | IRT ~ ID + B + C + B*C | 4.4888 | 0.64% |  |  |

S1 Table shows the null model (Model 0) and the alternative model (Model 1) for the different model comparisons along with the respective Bayes Factors of the models (BF) and their errors as well as the Bayes Factors of the model comparisons (BF_10_) with their respective errors in the object detection task of experiment 1 if only the first 68 subjects are included. Abbreviations: IRT = individual reaction times, ID = subject intercept, C = Reality Congruency, B = Belief.

**S2 Table. Bayes Factors for the model comparisons in the action detection task of exp. 1 for n=86.**

|  | Model 0 (1st row) and Model 1 (2nd row) | BF | Error | BF10 | Error |
| --- | --- | --- | --- | --- | --- |
| Belief | IRT ~ ID + C | 0.2154 | 0.86% | 0.1551 | 1.14% |
|  | IRT ~ ID + C + B | 0.0334 | 0.76% |  |  |
| Congruency | IRT ~ ID + B | 0.1555 | 0.27% | 0.2147 | 0.80% |
|  | IRT ~ ID + B + C | 0.0334 | 0.76% |  |  |
| Belief * Congruency | IRT ~ ID + B + C | 0.0334 | 0.76% | 4.84 | 1.83% |
|  | IRT ~ ID + B + C + B*C | 0.1618 | 1.67% |  |  |

S2 Table shows the null model (Model 0) and the alternative model (Model 1) for the different model comparisons along with the respective Bayes Factors of the models (BF) and their errors as well as the Bayes Factors of the model comparisons (BF_10_) with their respective errors in the action detection task of experiment 1 if only the first 86 subjects are included. Abbreviations: IRT = individual reaction times, ID = subject intercept, C = Reality Congruency, B = Belief.

**S2 Fig. Sequential testing in experiment 1.**

*
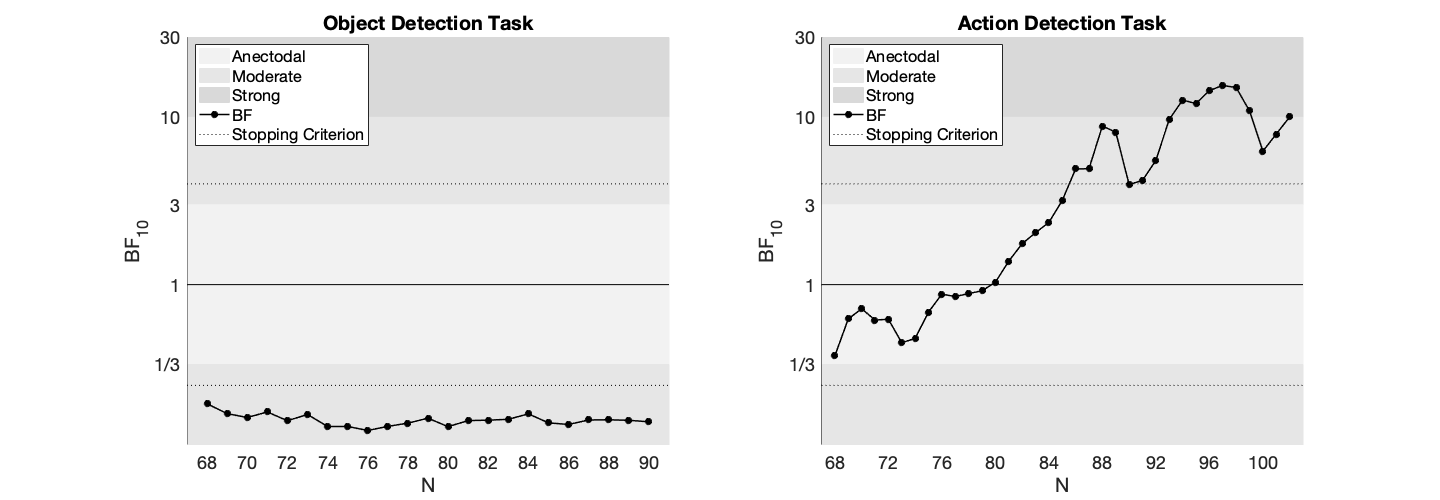
*

Development of the Bayes Factor of the interaction between *belief* and *reality congruency* in experiment 1 after the minimal number of participants, i.e. 68, was reached. Left Side: object detection task, right side: action detection task.

S4 Experiment 1: Secondary analysis ARTs

As a secondary analysis, we conducted two repeated measures Bayesian ANOVAs on the average response times (ARTs) using default priors with the within-subject factors *belief* and *reality congruency* and the between-subject covariates *initial-belief* (i.e. the first trial was a true belief trial vs the first trial was a false belief trial) and *initial-congruency* (i.e. the first trial was a reality congruent trial vs the first trial was a reality incongruent trial). We initially preregistered an ANCOVAs, but since our covariates were not metric, we ran ANOVAs instead. In order not to make our models unnecessarily complex, we only included the most meaningful interactions, i.e. an interaction between *belief* and *reality congruency,* an interaction between *belief* and *initial belief* as well as an interaction between *reality congruency* and *initial congruency*. Results are summarized in S3 Table.

For the object detection task, our model comparisons provided moderate evidence against a main effect of *belief* (BF_10_=0.12) and extreme evidence for a main effect of *reality congruency* (BF_10_=74507). For the *initial belief* and *initial congruency* the Bayes Factors remained inconclusive (BF_10_=0.44 for *initial belief* and BF_10_=0.61 for *initial congruency*). Moreover, our results yielded moderate evidence against all included interactions (BF_10_=0.18 between *reality congruency* and *belief*, BF_10_=0.27 between *initial-belief* and *belief*, BF_10_=0.17 between *initial-congruency* and *reality congruency*).

For the action detection task, our analysis provided moderate evidence against a main effect of *belief* (BF_10_=0.18), while the Bayes Factors of the main remaining main effects were inconclusive (BF_10_=0.44 for *reality congruency*, BF_10_=0.45 for *initial belief*, BF_10_=0.49 for *initial congruency*). For the interaction between *reality congruency* and *belief*, our model comparisons yielded strong evidence (BF_10_=24) while they provided moderate evidence against the remaining interactions (BF_10_=0.19 for the interaction between *initial belief* and *belief,* BF_10_=0.21 for the interaction between *initial congruency* and *reality congruency*).

**S3 Table. Bayes Factors for the model comparisons of experiment 1 for ARTs.**

|  |  | Object Detection Task | | | | Action Detection Task | | | |
| --- | --- | --- | --- | --- | --- | --- | --- | --- | --- |
|  | Model 0 (1st row) and Model 1 (2nd row) | BF | Error % | BF1/BF0 | Error % | BF | Error % | BF1/BF0 | Error % |
| Belief | ART ~ ID + IB + IC + C | 1.96E+80 | 0.61% | 0.1150 | 0.97% | 2.84E+105 | 0.75% | 0.1751 | 1.19% |
|  | ART ~ ID + IB + IC + C + B | 2.26E+79 | 0.76% |  |  | 4.97E+104 | 0.93% |  |  |
| Congruency | ART ~ ID + IB + IC + B | 3.03E+74 | 0.67% | 74507 | 1.01% | 1.14E+105 | 0.76% | 0.4349 | 1.20% |
|  | ART ~ ID + IB + IC + B + C | 2.26E+79 | 0.76% |  |  | 4.97E+104 | 0.93% |  |  |
| Initial Belief | ART ~ ID + IC + B + C | 5.09E+79 | 0.40% | 0.4427 | 0.86% | 1.11E+105 | 0.81% | 0.4485 | 1.23% |
|  | ART ~ ID + IC + B + C + IB | 2.26E+79 | 0.76% |  |  | 4.97E+104 | 0.93% |  |  |
| Initial Congruency | ART ~ ID + IB + B + C | 3.71E+79 | 0.68% | 0.6084 | 1.02% | 1.02E+105 | 0.82% | 0.4883 | 1.24% |
|  | ART ~ ID + IB + B + C + IC | 2.26E+79 | 0.76% |  |  | 4.97E+104 | 0.93% |  |  |
| Initial Belief*Belief | ART ~ ID + IB + IC + B + C + IC*C + B*C | 6.98E+77 | 1.15% | 0.2653 | 1.79% | 2.35E+105 | 1.27% | 0.1875 | 1.99% |
|  | ART ~ ID + IB + IC + B + C + IB*B + IC*C + B*C | 1.85E+77 | 1.37% |  |  | 4.41E+104 | 1.53% |  |  |
| Initial Cong. *Congruency | ART ~ ID + IB + IC + B + C + IB*B + B*C | 1.12E+78 | 1.12% | 0.1656 | 1.77% | 2.08E+105 | 1.28% | 0.2121 | 2.00% |
|  | ART ~ ID + IB + IC + B + C + IB*B + IC*C + B*C | 1.85E+77 | 1.37% |  |  | 4.41E+104 | 1.53% |  |  |
| Belief * Congruency | ART ~ ID + IB + IC + B + C + IB*B + IC*C | 1.03E+78 | 1.14% | 0.1792 | 1.78% | 1.86E+103 | 1.37% | 23.78 | 2.06% |
|  | ART ~ ID + IB + IC + B + C + IB*B + IC*C + B*C | 1.85E+77 | 1.37% |  |  | 4.41E+104 | 1.53% |  |  |

S3 Table shows the null model (Model 0) and the alternative model (Model 1) for the different model comparisons along with the respective Bayes Factors of the models (BF) and their errors as well as the Bayes Factors of the model comparisons (BF_10_) with their respective errors in experiment 1 for a repeated-measures ANOVA on average reaction times. Abbreviations: IRT = individual reaction times, ID = subject intercept, C = Reality Congruency, B = Belief

S5 Experiment 2: Violation of normality

Quantile-quantile (Q-Q) plots indicated right skewed distributions for the average as well as the individual response times in the implicit and the action detection task that deviated from normality. After log-transformation we still observed some deviations from normality that were mainly driven by a few outliers (see S3 Fig and S4 Fig). To assure that these outliers did not drive our results, we repeated all analyses after removing elements that were more than 1.5 interquartile ranges above the upper or below the lower quartile (see S4 Table). Overall, this analysis yielded the same results for our main variables of interest (*belief*, *reality congruency*, interaction). Minor differences are discussed in the following.

**S3 Fig. Q-Q-Plots of the log-transformed IRTs in experiment 2.**


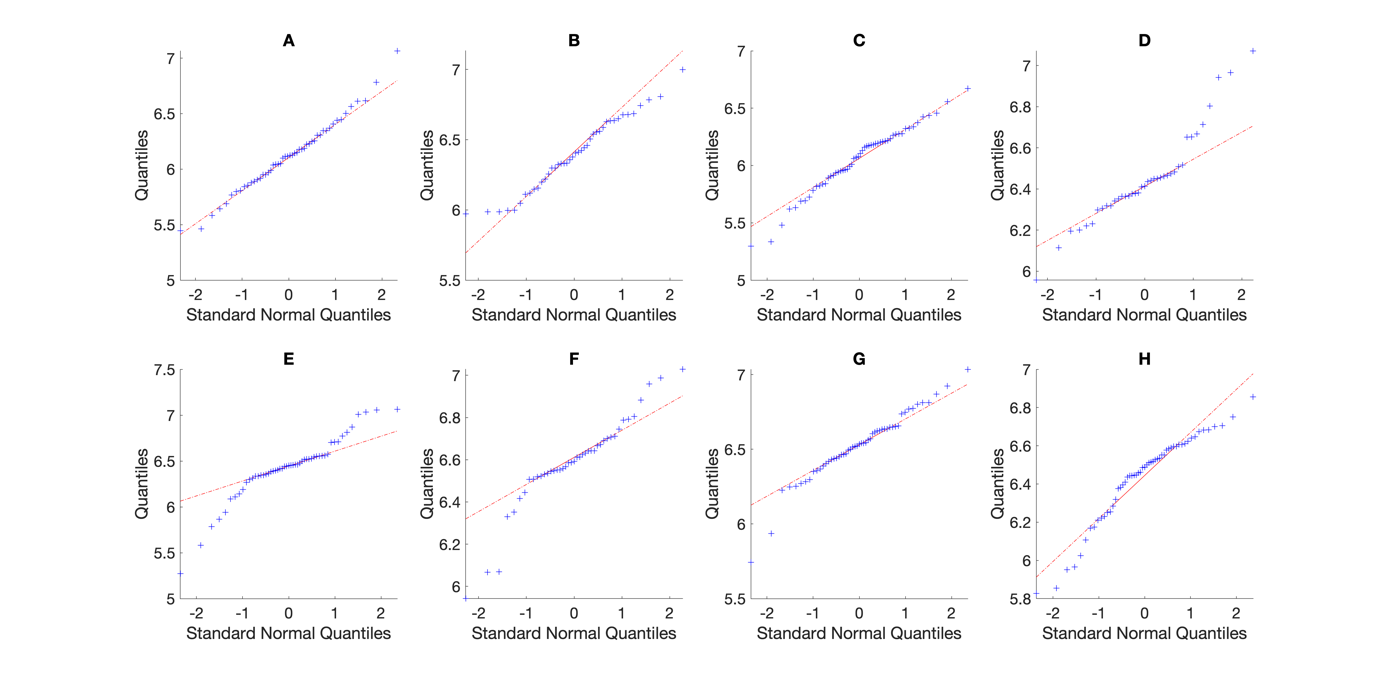


Quantiles of the log-transformed individual response times in the first two trials plotted against the theoretical quantile values from a normal distribution. A: log-IRTs in true belief reality congruent trials in the object detection task, B: log-IRTs in true belief reality incongruent trials in the object detection task, C: log-IRTs in false belief reality congruent trials in the object detection task, D: log-IRTs in false belief reality incongruent trials in the object detection task, E: log-IRTs in true belief reality congruent trials in the action detection task, F: log-IRTs in true belief reality incongruent trials in the action detection task, G: log-IRTs in false belief reality congruent trials in the action detection task, H: log-IRTs in false belief reality incongruent trials in the action detection task.

**S4 Fig. Q-Q-Plots of the log-transformed ARTs in experiment 2.**


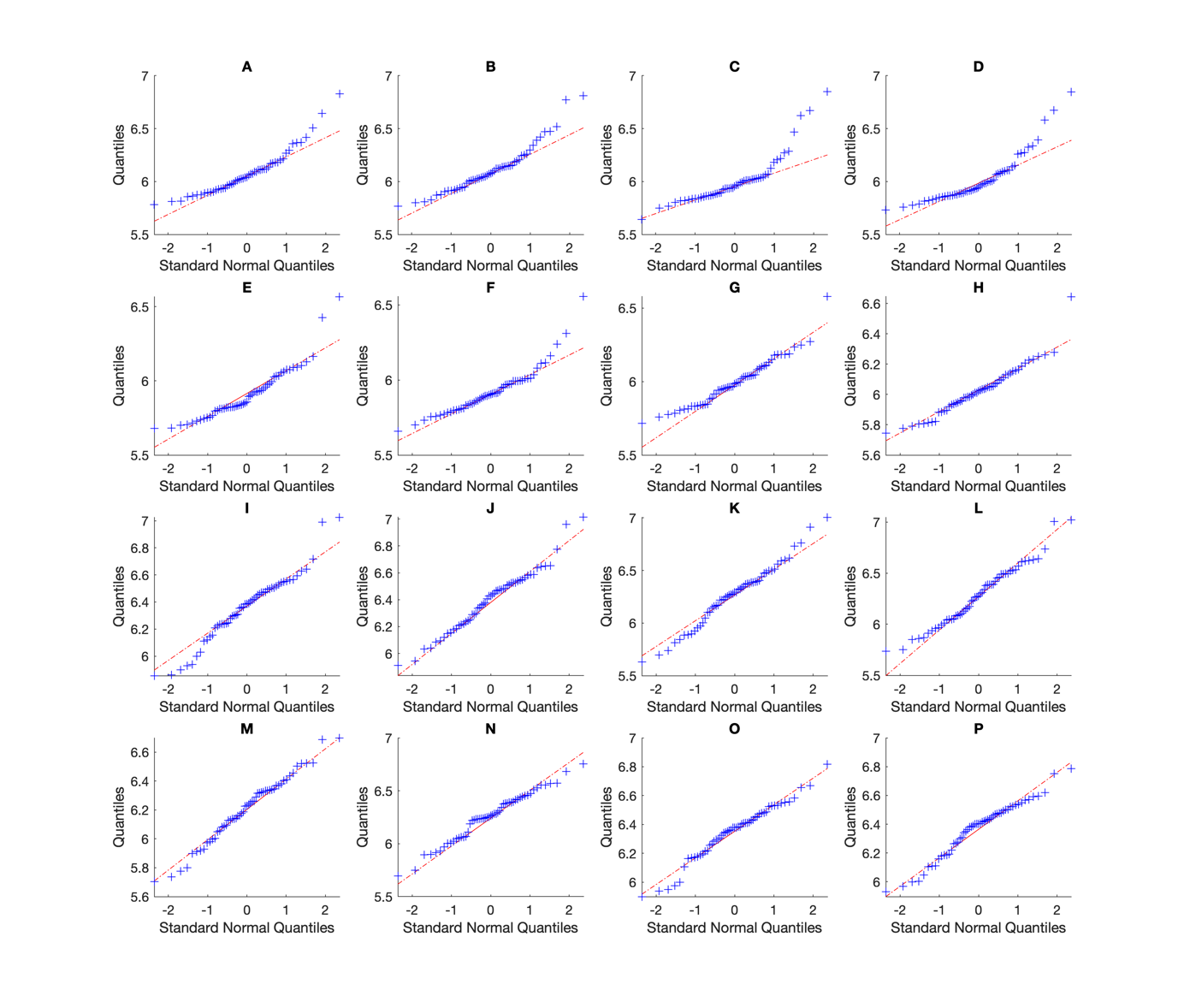
 Quantiles of the log-transformed average response times plotted against the theoretical quantile values from a normal distribution. A-H: log-ARTs in the object detection task, I-P: log-ARTs in the action detection task, A-D and I-L: data from subjects who completed the true belief version before the false belief version of the tasks, E-H and M-P: data from subjects who completed the false belief version before the true belief version of the tasks, first row (A, E, I, M): true belief and reality congruent trials, second row (B, F, J, N): true belief and reality incongruent trials, third row (C, G, K, O): false belief and reality congruent trials, forth row (D, H, L, P): false belief and reality incongruent trials.

**S4 Table. Bayes Factors for the model comparisons of experiment 2 without outliers.**

|  |  |  | Object Detection Task | | | | Action Detection Task | | | |
| --- | --- | --- | --- | --- | --- | --- | --- | --- | --- | --- |
|  |  | Model 0 (1st row) and Model 1 (2nd row) | BF | Error | BF10 | Error | BF | Error | BF10 | Error |
| First Trials | Belief | IRT ~ ID + C | 1.42E+13 | 1.31% | 0.2596 | 1.35% | 0.1637 | 0.23% | 0.2859 | 0.62% |
|  |  | IRT ~ ID + C + B | 3.70E+12 | 0.31% |  |  | 0.0468 | 0.58% |  |  |
|  | Congruency | IRT ~ ID + B | 0.2555 | 0.26% | 1.45E+13 | 0.41% | 0.2818 | 0.48% | 0.1661 | 0.75% |
|  |  | IRT ~ ID + B + C | 3.70E+12 | 0.31% |  |  | 0.0468 | 0.58% |  |  |
|  | Belief * Congruency | IRT ~ ID + B + C | 3.70E+12 | 0.31% | 0.2943 | 1.50% | 0.0468 | 0.58% | 12597.53 | 0.84% |
|  |  | IRT ~ ID + B + C + B*C | 1.09E+12 | 1.47% |  |  | 589.68 | 0.61% |  |  |
| All Trials | Belief | ART ~ ID + C + IB | 1.3602 | 1.50% | 0.1292 | 1.90% | 3.2898 | 1.30% | 0.1910 | 2.10% |
|  |  | ART ~ ID + C + IB + B | 0.1757 | 1.17% |  |  | 0.6283 | 1.65% |  |  |
|  | Congruency | ART ~ ID + B + IB | 0.0291 | 1.08% | 6.04 | 1.59% | 0.1613 | 0.87% | 3.8966 | 1.87% |
|  |  | ART ~ ID + B + IB + C | 0.1757 | 1.17% |  |  | 0.6283 | 1.65% |  |  |
|  | Initial-Belief | ART ~ ID + B + C | 0.7959 | 0.48% | 0.2207 | 1.26% | 0.7660 | 0.91% | 0.8202 | 1.88% |
|  |  | ART ~ ID + B + C + IB | 0.1757 | 1.17% |  |  | 0.6283 | 1.65% |  |  |
|  | Initial-Belief * Belief | ART ~ ID + B + C + IB + C*IB + B*C | 0.0056 | 1.07% | 0.1670 | 1.62% | 0.0464 | 1.95% | 0.1497 | 2.81% |
|  |  | ART ~ ID + B + C + IB + C*IB + B*C + B*IB | 0.0009 | 1.21% |  |  | 0.0070 | 2.02% |  |  |
|  | Initial-Belief * Congruency | ART ~ ID + B + C + IB + B*IB + B*C | 0.0042 | 1.16% | 0.2228 | 1.68% | 0.0461 | 1.90% | 0.1507 | 2.78% |
|  |  | ART ~ ID + B + C + IB + B*IB + B*C + C*IB | 0.0009 | 1.21% |  |  | 0.0070 | 2.02% |  |  |
|  | Belief * Congruency | ART ~ ID + B + C + IB + B*IB + C*IB | 0.0060 | 1.20% | 0.1565 | 1.71% | 0.0150 | 2.59% | 0.4640 | 3.29% |
|  |  | ART ~ ID + B + C + IB + B*IB + C*IB + B*C | 0.0009 | 1.21% |  |  | 0.0070 | 2.02% |  |  |

S4 Table shows the null model (Model 0) and the alternative model (Model 1) for the different model comparisons along with the respective Bayes Factors of the models (BF) and their errors as well as the Bayes Factors of the model comparisons (BF_10_) with their respective errors in experiment 2 after outlier removal (i.e. values that were more than 1.5 interquartile ranges above the upper or below the lower quartile). Abbreviations: IRT = individual reaction times, ID = subject intercept, C = Reality Congruency, B = Belief.

For the analysis of IRTs, removing outliers led to moderate evidence against an interaction between *belief* and *reality congruency* for the object detection task (without outliers BF_10_=0.29). For the corresponding analysis with outliers, the Bayes Factor of this interaction had remained inconclusive (with outliers BF_10_=1.1).

For the analysis of ARTs, the Bayes Factors of all single models became very small (BFs<1.4 for the object detection task, BFs < 3.3 for the action detection task) indicating less evidence for the models themselves and, thus, less informative value for the model comparisons. Nevertheless, they pointed into the same direction as before for our main variables of interest (*belief*, *reality congruency*, interaction between them). Major differences could only be observed with respect to the covariate *initial-belief*. For the object detection task, the model comparisons provided moderate evidence against a main effect of *initial-belief* (without outliers: BF_10_=0.22) instead of moderate evidence for an effect (with outliers: BF_10_=4). For both the implicit and the action detection task, the analysis without outliers provided moderate evidence against an interaction between *initial-belief* and *belief* (without outliers: BF_10_=0.17 for the object detection task and BF_10_=0.15 for the action detection task) instead of extreme evidence for an interaction (with outliers: BF_10_=1.147761E+37 for the implicit and BF_10_=3.760997E+26 for the action detection task). This could indicate that the main effect of the covariate *initial-belief* and the interaction with *belief* (that is, in fact, a simple learning effect as stated in the discussion of experiment 1) are mainly driven by outliers. Still, considering the low Bayes Factors for the single models, this conclusion needs to be considered with caution.

S6 Experiment 2: Sequential testing

In experiment 2, the predefined stopping criterion (i.e. Bayes Factor above 4 or below 0.25 for the interaction between *reality congruency* and *belief*) was already reached after collecting valid data of 69 participants for the object detection task (see S5 Fig left). Only including data of those participants let to inconclusive Bayes Factors for a main effect of *congruency* and *initial-belief*. Other than that, it did not alter the results (see S5 Table).

For the action detection task, the respective Bayes factor was not yet conclusive at this point, so we continued to collect data (see S5 Fig). Although the stopping criterion was still not reached after n=115 participants for the action detection task, data collection was stopped at this point to obtain similar sample sizes in both experiment 1 and experiment 2.

**S5 Table. Bayes Factors for the model comparisons of exp. 2 for the object detection task for n=69.**

|  |  | Model 0 (1st row) and Model 1 (2nd row) | BF | Error | BF10 | Error |
| --- | --- | --- | --- | --- | --- | --- |
| First Trials | Belief | IRT ~ ID + C | 5063208 | 1.15% | 0.3421 | 1.41% |
|  |  | IRT ~ ID + C + B | 1731917 | 0.82% |  |  |
|  | Congruency | IRT ~ ID + B | 0.3063 | 0.69% | 5653441 | 1.07% |
|  |  | IRT ~ ID + B + C | 1731917 | 0.82% |  |  |
|  | Belief * Congruency | IRT ~ ID + B + C | 1731917 | 0.82% | 2.11 | 0.89% |
|  |  | IRT ~ ID + B + C + B*C | 3661795 | 0.33% |  |  |
| All Trials | Belief | ART ~ ID + C + IB | 1.72 | 2.78% | 0.1361 | 4.20% |
|  |  | ART ~ ID + C + IB + B | 0.2335 | 3.15% |  |  |
|  | Congruency | ART ~ ID + B + IB | 0.1318 | 1.69% | 1.77 | 3.57% |
|  |  | ART ~ ID + B + IB + C | 0.2335 | 3.15% |  |  |
|  | Initial-Belief | ART ~ ID + B + C | 0.2331 | 0.85% | 1.00 | 3.26% |
|  |  | ART ~ ID + B + C + IB | 0.2335 | 3.15% |  |  |
|  | Initial-Belief *Belief | ART ~ ID + B + C + IB + C*IB + B*C | 0.0075 | 2.77% | 3.13E+23 | 3.76% |
|  |  | ART ~ ID + B + C + IB + C*IB + B*C + B*IB | 2.34E+21 | 2.55% |  |  |
|  | Initial-Belief *Congruency | ART ~ ID + B + C + IB + B*IB + B*C | 1.34E+22 | 2.70% | 0.1746 | 3.71% |
|  |  | ART ~ ID + B + C + IB + B*IB + B*C + C*IB | 2.34E+21 | 2.55% |  |  |
|  | Belief * Congruency | ART ~ ID + B + C + IB + B*IB + C*IB | 1.41E+22 | 4.36% | 0.1656 | 5.05% |
|  |  | ART ~ ID + B + C + IB + B*IB + C*IB + B*C | 2.34E+21 | 2.55% |  |  |

S5 Table shows the null model (Model 0) and the alternative model (Model 1) for the different model comparisons along with the respective Bayes Factors of the models (BF) and their errors as well as the Bayes Factors of the model comparisons (BF_10_) with their respective errors in experiment 2 for the object detection task if only data of the first 69 participants is included. Abbreviations: IRT = individual reaction times, ID = subject intercept, C = Reality Congruency, B = Belief.

**S5 Fig. Sequential testing in experiment 2.
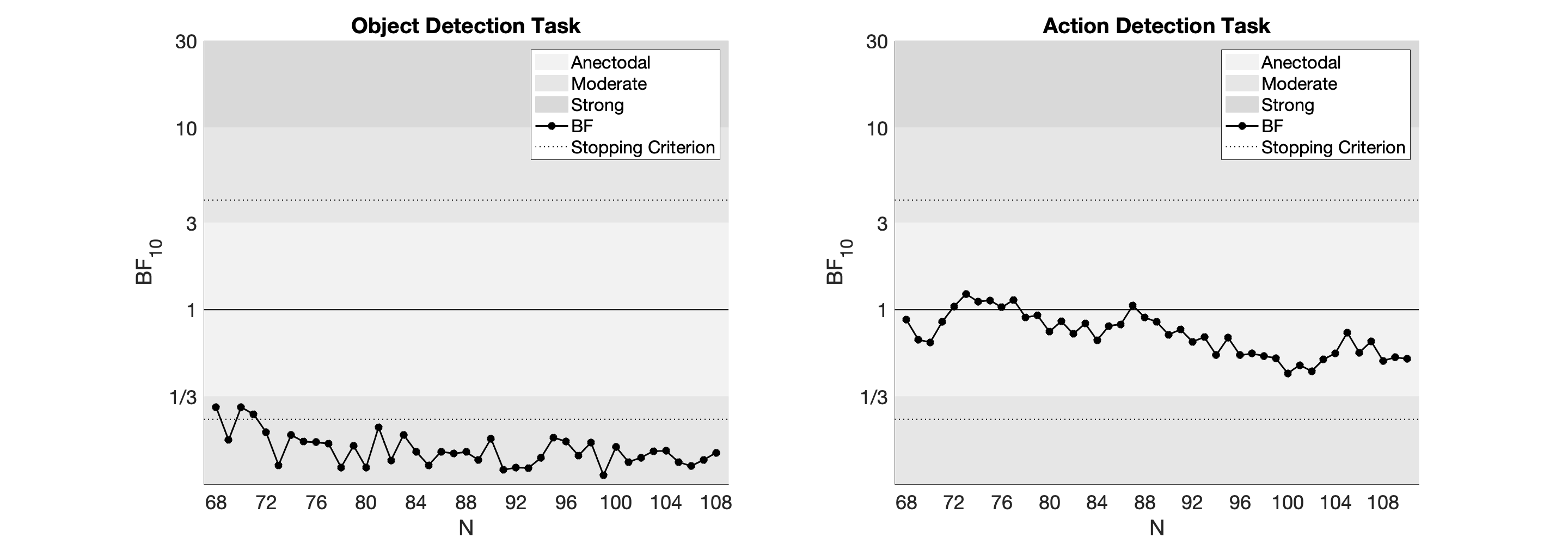
**

Development of the Bayes Factor of the interaction between *belief* and *reality congruency* after the minimal number of participants, i.e. 68, was reached. Left Side: object detection task, right side: action detection task.

S7 Experiment 2: Secondary analysis first block

Since the agent’s belief in the first experimental block might have influenced the participant’s interpretation of the second experimental block, we additionally performed a mixed ANOVA with the within-subjects factor *reality* *congruency* and the between-subjects factor *belief* including only the data of the first experimental block for each task.

**S6 Table. Bayes Factors for the model comparisons in experiment 1 including only the data of the first block of the object detection task.**

|  | Model 0 (1st row) and Model 1 (2nd row) | BF | Error % | BF1/BF0 | Error % |
| --- | --- | --- | --- | --- | --- |
| Belief | IRT ~ ID + C | 10233.5000 | 0.32% | 2.0557 | 2.31% |
|  | IRT ~ ID + C + B | 21037.4000 | 2.29% |  |  |
| Congruency | IRT ~ ID + B | 2.1202 | 4.85% | 9922.3760 | 5.36% |
|  | IRT ~ ID + B + C | 21037.4000 | 2.29% |  |  |
| Belief * Congruency | IRT ~ ID + B + C | 21037.4000 | 2.29% | 0.1918 | 2.85% |
|  | IRT ~ ID + B + C + B*C | 4034.8490 | 1.69% |  |  |

S6 Table shows the null model (Model 0) and the alternative model (Model 1) for the different model comparisons along with the respective Bayes Factors of the models (BF) and their errors as well as the Bayes Factors of the model comparisons (BF_10_) with their respective errors in the action detection task of experiment 1 if only the data of the first experimental block of the object detection task is included. Abbreviations: IRT = individual reaction times, ID = subject intercept, C = Reality Congruency, B = Belief.

For the object detection task, our model comparisons remain inconclusive for a main effect of belief (BF_10_=2), they provide extreme evidence for a main effect of congruency (BF_10_=9922) and moderate evidence against an interaction between the two (BF_10_=0.19) (see S6 Table). For the action detection task, our analyses also remain inconclusive for a main effect of belief (BF_10_=0.49), they provide moderate evidence for a main effect of congruency (BF_10_=4) and moderate evidence against an interaction between the two (BF_10_=0.33) (see S7 Table).

**S7 Table. Bayes Factors for the model comparisons in experiment 1 including only the data of the first block of the action detection task.**

|  | Model 0 (1st row) and Model 1 (2nd row) | BF | Error % | BF1/BF0 | Error % |
| --- | --- | --- | --- | --- | --- |
| Belief | IRT ~ ID + C | 3.8743 | 0.64% | 0.491 | 0.85% |
|  | IRT ~ ID + C + B | 1.9021 | 0.57% |  |  |
| Congruency | IRT ~ ID + B | 0.4773 | 0.48% | 3.9849 | 0.74% |
|  | IRT ~ ID + B + C | 1.9021 | 0.57% |  |  |
| Belief * Congruency | IRT ~ ID + B + C | 1.9021 | 0.57% | 0.3299 | 0.89% |
|  | IRT ~ ID + B + C + B*C | 0.6276 | 0.69% |  |  |

S7 Table shows the null model (Model 0) and the alternative model (Model 1) for the different model comparisons along with the respective Bayes Factors of the models (BF) and their errors as well as the Bayes Factors of the model comparisons (BF_10_) with their respective errors in the action detection task of experiment 1 if only the data of the first experimental block of the action detection task is included. Abbreviations: IRT = individual reaction times, ID = subject intercept, C = Reality Congruency, B = Belief.

S8 Experiment 2: Secondary analysis IRTs

To control for repetition effects, we additionally analyzed the individual responses using a Bayesian linear mixed model (LMM) with default priors including the factors *belief*, *reality* *congruency*, *subject*, *trial order*, and side of *button press*. In order not to make the models unnecessarily complex, we only included the more meaningful interactions, i.e. an interaction between *belief* and *reality* *congruency*, between *belief* and *trial order* as well as between *reality congruency* and *trial order*. For the within-subject factors *belief*, *congruency* and side of *button press,* random slopes were included for the main effects and interactions. A random slope allows an interaction between subject and factor (or interaction, respectively). That is, the model accounts for the fact that a difference observed for a factor may be different for different participants. We did not include random slopes in our analyses of ARTs, since data aggregation reduces or even eliminates the possibility to detect random slopes (Doorn, Aust, Haaf, Angelika, & Wagenmakers, n.d.). For the IRT analyses of the first two trials only, fitting random slopes was impossible, since there was only one value in each cell.

For linear mixed models, checking the assumption of normality in all different subgroups of the model is often infeasible. Alternatively, the assumption of normality can be examined by looking at the distribution of residuals. In the present case, the distribution of residuals indicated deviations from normality even for the log-transformed data (see S6 Fig). These deviations were mainly driven by outliers as in the other analyses. Therefore, we decided to still move forward with our original analysis plans.

**S6 Fig. Q-Q-Plot of the residuals for the GLM.**


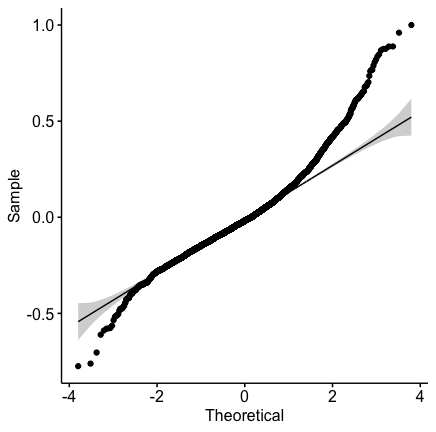

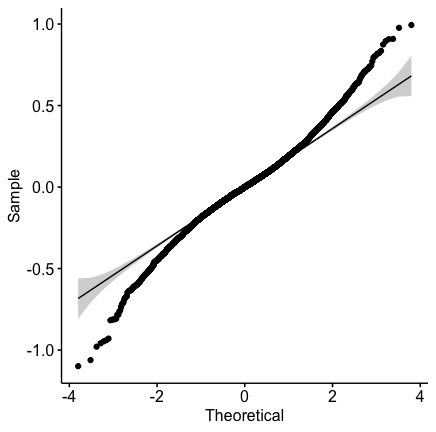


Quantiles of the residuals of the general linear model plotted against the theoretical quantile values from a normal distribution. Left: Object detection task, Right: Action detection task.

For the object detection task, the model comparisons provided strong evidence against a main effect of the factor *belief* (BF_10_=0.06), moderate evidence for a main effect of *reality congruency* (BF_10_=5) and extreme evidence for a main effect of *trial order* and side of the *button press* (for both BF_10_ >1,000,000, see S8 Table). Furthermore, there was strong evidence against an interaction between *reality congruency* and *belief* (BF_10_=0.05) as well as against an interaction between *belief* and *trial order* (BF_10_=0.08), while the Bayes Factor remained inconclusive for the interaction between *reality congruency* and *trial order* (BF_10_=1.58).

For the action detection task, the model comparisons yielded moderate evidence against a main effect of *belief* (BF_10_=0.12) and extreme evidence for a main effect of *reality congruency* (BF_10_=515) as well as *trial order* (BF_10_>1,000,000), while evidence remained inconclusive for side of the *button press* (BF_10_ =2.85, see S9 Table). In addition, there was moderate evidence against an interaction between *reality congruency* and *belief* (BF_10_=0.2) as well as against an interaction between *reality congruency* and *trial order* (BF_10_=0.27), while the Bayes Factor remained inconclusive for the interaction between *belief* and *trial order* (BF_10_=0.82).

For both tasks, the results of the analyses of IRTs are completely in line with the results of the analyses of ARTs.

**S8 Table. Bayes Factors for the model comparisons of the object detection task in experiment 2 for the analysis of IRTs in a GLM.**

|  | Model 0 (1st row) and Model 1 (2nd row) | BF | Err % | BF1/BF0 | Err % |
| --- | --- | --- | --- | --- | --- |
| Belief | IRT ~ ID + B*ID + C*ID + C + O + BP*ID + BP | 1.288368e+1127 | 1.68% | 0.0628 | 1.95% |
|  | IRT ~ ID + B*ID + B + C*ID + C + O + BP*ID + BP | 8.090675e+1125 | 0.98% |  |  |
| Congruency | IRT ~ ID + B*ID + B + C*ID + O + BP*ID + BP | 1.516849e+1125 | 1.52% | 5.3339 | 1.81% |
|  | IRT ~ ID + B*ID + B + C*ID + C + O + BP*ID + BP | 8.090675e+1125 | 0.98% |  |  |
| Order | IRT ~ ID + B*ID + B + C*ID + C + BP*ID + BP | 3.913024e+1049 | 2.75% | 2.07E+76 | 2.92% |
|  | IRT ~ ID + B*ID + B + C*ID + C + O + BP*ID + BP | 8.090675e+1125 | 0.98% |  |  |
| Button Press | IRT ~ ID + B*ID + B + C*ID + C + O + BP*ID | 7.807802e+1115 | 1.74% | 1.04E+10 | 2.00% |
|  | IRT ~ ID + B*ID + B + C*ID + C + O + BP*ID + BP | 8.090675e+1125 | 0.98% |  |  |
| Belief*Order | IRT ~ ID + B*ID + B + C*ID+ C + O + BP*ID + BP + C*O + B*C*ID + B*C | 2.325543e+1115 | 2.09% | 0.0756 | 3.88% |
|  | IRT ~ ID + B*ID + B + C*ID+ C + O + BP*ID + BP + C*O + B*O + B*C*ID + B*C | 1.757999e+1114 | 3.27% |  |  |
| Congruency*Order | IRT ~ ID + B*ID + B + C*ID+ C + O + BP*ID + BP + B*O + B*C*ID + B*C | 1.115472e+1114 | 0.99% | 1.5760 | 3.41% |
|  | IRT ~ ID + B*ID + B + C*ID+ C + O + BP*ID + BP + C*O + B*O + B*C*ID + B*C | 1.757999e+1114 | 3.27% |  |  |
| Belief* Congruency | IRT ~ ID + B*ID + B + C*ID+ C + O + BP*ID + BP + C*O + B*O + B*C*ID | 3.445112e+1115 | 1.22% | 0.0510 | 3.49% |
|  | IRT ~ ID + B*ID + B + C*ID+ C + O + BP*ID + BP + C*O + B*O + B*C*ID + B*C | 1.757999e+1114 | 3.27% |  |  |

S8 Table shows the null model (Model 0) and the alternative model (Model 1) for the different model comparisons along with the respective Bayes Factors of the models (BF) and their errors as well as the Bayes Factors of the model comparisons (BF_10_) with their respective errors in experiment 2 if individual reaction times are analyzed with a general linear model (i.e. values that were more than 1.5 interquartile ranges above the upper or below the lower quartile). Abbreviations: IRT = individual reaction times, ID = subject intercept, C = Reality Congruency, B = Belief, BP = button press, O = trial order.

**S9 Table. Bayes Factors for the model comparisons of the action detection task in experiment 2 for the analysis of IRTs in a GLM.**

|  | Model 0 (1st row) and Model 1 (2nd row) | BF | Err % | BF1/BF0 | Err % |
| --- | --- | --- | --- | --- | --- |
| Belief | IRT ~ ID + B*ID + C*ID + C + O + BP*ID + BP | 2.762259e+1076 | 0.99% | 0.1208 | 2.21% |
|  | IRT ~ ID + B*ID + B + C*ID + C + O + BP*ID + BP | 3.335455e+1075 | 1.98% |  |  |
| Congruency | IRT ~ ID + B*ID + B + C*ID + O + BP*ID + BP | 6.478163e+1072 | 1.23% | 514.88 | 2.33% |
|  | IRT ~ ID + B*ID + B + C*ID + C + O + BP*ID + BP | 3.335455e+1075 | 1.98% |  |  |
| Order | IRT ~ ID + B*ID + B + C*ID + C + BP*ID + BP | 1.398483e+1010 | 1.84% | 2.39E+65 | 2.71% |
|  | IRT ~ ID + B*ID + B + C*ID + C + O + BP*ID + BP | 3.335455e+1075 | 1.98% |  |  |
| Button Press | IRT ~ ID + B*ID + B + C*ID + C + O + BP*ID | 1.17148e+1075 | 1.34% | 2.8472 | 2.40% |
|  | IRT ~ ID + B*ID + B + C*ID + C + O + BP*ID + BP | 3.335455e+1075 | 1.98% |  |  |
| Belief*Order | IRT ~ ID + B*ID + B + C*ID+ C + O + BP*ID + BP + C*O + B*C*ID + B*C | 6.166428e+1102 | 1.78% | 0.8216 | 2.66% |
|  | IRT ~ ID + B*ID + B + C*ID+ C + O + BP*ID + BP + C*O + B*O + B*C*ID + B*C | 5.066427e+1102 | 1.97% |  |  |
| Congruency*Order | IRT ~ ID + B*ID + B + C*ID+ C + O + BP*ID + BP + B*O + B*C*ID + B*C | 1.853681e+1103 | 2.10% | 0.2733 | 2.88% |
|  | IRT ~ ID + B*ID + B + C*ID+ C + O + BP*ID + BP + C*O + B*O + B*C*ID + B*C | 5.066427e+1102 | 1.97% |  |  |
| Belief* Congruency | IRT ~ ID + B*ID + B + C*ID+ C + O + BP*ID + BP + C*O + B*O + B*C*ID | 2.584997e+1103 | 2.94% | 0.1960 | 3.54% |
|  | IRT ~ ID + B*ID + B + C*ID+ C + O + BP*ID + BP + C*O + B*O + B*C*ID + B*C | 5.066427e+1102 | 1.97% |  |  |

S9 Table shows the null model (Model 0) and the alternative model (Model 1) for the different model comparisons along with the respective Bayes Factors of the models (BF) and their errors as well as the Bayes Factors of the model comparisons (BF_10_) with their respective errors in experiment 2 if individual reaction times are analyzed with a general linear model (i.e. values that were more than 1.5 interquartile ranges above the upper or below the lower quartile). Abbreviations: IRT = individual reaction times, ID = subject intercept, C = Reality Congruency, B = Belief, BP = button press, O = trial order.

S9 Supporting information: References

Doorn, J. Van, Aust, F., Haaf, J. M., Angelika, M., & Wagenmakers, E. (n.d.). *Bayes Factors for Mixed Models*. 1–32.

El Kaddouri, R., Bardi, L., De Bremaeker, D., Brass, M., & Wiersema, J. R. (2019). Measuring spontaneous mentalizing with a ball detection task: putting the attention-check hypothesis by Phillips and colleagues (2015) to the test. *Psychological Research*, (0123456789). https://doi.org/10.1007/s00426-019-01181-7

Lakens, D., & Caldwell, A. (2019). *Simulation-Based Power-Analysis for Factorial ANOVA Designs*. 1–11. https://doi.org/10.31234/osf.io/baxsf
