# Supplementary material for: Evidence against implicit belief processing in a blindfold task
Source: PLoS One. 2023 Nov 13;18(11):e0294136. doi: 10.1371/journal.pone.0294136 (PMC10642834; doi:10.1371/journal.pone.0294136)
Supplement: Attachment — Submitted filename: Response_to_Reviewers.docx [file pone.0294136.s002.docx]

**Response to the editor**

1. Please ensure that your manuscript meets PLOS ONE's style requirements, including those for file naming. The PLOS ONE style templates can be found at

<https://journals.plos.org/plosone/s/file?id=wjVg/PLOSOne_formatting_sample_main_body.pdf> and

<https://journals.plos.org/plosone/s/file?id=ba62/PLOSOne_formatting_sample_title_authors_affiliations.pdf>.

We have ensured that the manuscript meets all style requirements.

2. We note that you have stated that you will provide repository information for your data at acceptance. Should your manuscript be accepted for publication, we will hold it until you provide the relevant accession numbers or DOIs necessary to access your data. If you wish to make changes to your Data Availability statement, please describe these changes in your cover letter and we will update your Data Availability statement to reflect the information you provide.

We will ensure to provide repository information for our data upon acceptance.

3. We note that Figure 1 includes an image of a [patient / participant / in the study].

As per the PLOS ONE policy (<http://journals.plos.org/plosone/s/submission-guidelines#loc-human-subjects-research>) on papers that include identifying, or potentially identifying, information, the individual(s) or parent(s)/guardian(s) must be informed of the terms of the PLOS open-access (CC-BY) license and provide specific permission for publication of these details under the terms of this license. Please download the Consent Form for Publication in a PLOS Journal (<http://journals.plos.org/plosone/s/file?id=8ce6/plos-consent-form-english.pdf>). The signed consent form should not be submitted with the manuscript, but should be securely filed in the individual's case notes. Please amend the methods section and ethics statement of the manuscript to explicitly state that the patient/participant has provided consent for publication: “The individual in this manuscript has given written informed consent (as outlined in PLOS consent form) to publish these case details”.

If you are unable to obtain consent from the subject of the photograph, you will need to remove the figure and any other textual identifying information or case descriptions for this individual.

Thank you for this note. We have gathered the signed consent form and amended a statement that the individual in the figure has given written informed consent (as outlined in PLOS consent form) to publish her photo.

4. Please review your reference list to ensure that it is complete and correct. If you have cited papers that have been retracted, please include the rationale for doing so in the manuscript text, or remove these references and replace them with relevant current references. Any changes to the reference list should be mentioned in the rebuttal letter that accompanies your revised manuscript. If you need to cite a retracted article, indicate the article’s retracted status in the References list and also include a citation and full reference for the retraction notice.

We have reviewed the reference list and added the following reference due to the comment of reviewer #2: Teufel, C., Alexis, D. M., Clayton, N. S., & Davis, G. (2010). Mental-state attribution drives rapid, reflexive gaze following. Attention, Perception, & Psychophysics, 72(3), 695-705.

**Response to reviewer #1:**

Reviewer #1: Summary: In this work, the authors want to see if humans exhibit altercentric bias. They study this through a modification of existing object detection task, by introducing a blindfold (which may or may not be opaque). This ensures the stimuli remains the same, which was not so in earlier work.The participants are familiarized with the blindfolds, and the agent's belief could therefore, be inferred based on participants' experience. The second study is to understand the relationship between egocentric bias and altercentric bias (the two are expected to be opposing). To do this, they employ an action detection task. They find that reaction times were driven by congruency (not belief; therefore no altercentric bias).

The authors have pre-registered the experiments, and the statistical analyses look fine to me.

The complete data is NOT yet available, but I see that the authors have stated they'd make it available on OSF upon acceptance.

I am not sure whether there is enough of a contribution or novelty here, but I don't have any issues with the experiments, analyses, or conclusion.

We thank the reviewer very much for their positive feedback.

**Response to reviewer #2:**

Reviewer #2: This paper addresses a central question about implicit theory of mind by addressing the existence of an altercentric bias crated by another person's false belief. The two experiments are very impressive and the data carefully analysed. Very impressive.
The main objective is to sharpen claims based on existing evidence. For this the authors combined the method pioneered by Kovacs et al (2010) with transparent/opaque goggles. An effect with this addition would show that the bias could not be due to the socio-attentional processes but must be due to a cognitively inferred belief. Indeed if the altercercentric bias could have been found under these conditions it would have been valuable evidence for its existence. But the evidence is strongly negative.
This creates a different problem of interpretation as the authors acknowledge in their General Discussion. One of these counterarguments is that inferring a person's visual access from which goggles the person is wearing is beyond implicit processing. So no wonder that no altercentric bias could be found under these conditions. This counterargument could be attenuated if there was evidence that implicit processing is possible with goggles. There was, I think to remember, such evidence in the context of automatic gaze following (Teufel et al 2013). The authors might want to sharpen their conclusions with the help of this work—provided no strong counterevidence has accumulated since.
Teufel, C., Alexis, D. M., Clayton, N. S., & Davis, G. (2010). Mental-state attribution drives rapid, reflexive gaze following. Attention, Perception, & Psychophysics, 72(3), 695-705.

We thank the reviewer very much for their enthusiastic feedback and their valuable input. As recommended, we included the argument in our discussion (page 32 line 730 and following):

*Parts of this complex process, such as deriving whether an agent can or cannot see based on previous experience with a blindfold, have been shown to occur on an automatic level in the simpler context of gaze following [39]. However, in the context of false belief situations, the process as a whole may have been too complex to function spontaneously.*

and also sharpened our conclusion (page 34 line 781-782):

*That is, we may spontaneously be influenced by the perspective of others if it can be grasped at an immediate perceptual level, such as in gaze following or visual perspective taking, but may not take their perspective or belief into account if it needs to be inferred in a cognitively more effortful way, for example, from one’s own experience about visual access*

We did not skip the entire argument, though, because we still believe that it is possible that inferring the belief of a person based on previous experience with a blindfold is cognitively more demanding than inferring whether or not a person can see through a blindfold in a simpler context like gaze following.
